# Examination of *Mycobacterium avium* subspecies *paratuberculosis* mixed genotype infections in dairy animals using a whole genome sequencing approach

**DOI:** 10.7717/peerj.2793

**Published:** 2016-12-14

**Authors:** Fraser W. Davidson, Christina Ahlstrom, Jeroen De Buck, Hugh G. Whitney, Kapil Tahlan

**Affiliations:** 1 Department of Biology, Memorial University of Newfoundland, St. John’s, NL, Canada; 2 Faculty of Veterinary Medicine, University of Calgary, Calgary, AB, Canada

**Keywords:** *M. avium* subsp. *paratuberculosis*, Genome sequencing, Mixed genotype infection

## Abstract

Many pathogenic mycobacteria are known to cause severe disease in humans and animals. *M. avium* subspecies *paratuberculosis* (*Map*) is the causative agent of Johne’s disease—a chronic wasting disease affecting ruminants such as cattle and sheep, responsible for significant economic losses in the dairy and beef industries. Due to the lack of treatment options or effective vaccines, mitigating losses can be difficult. In addition, the early stages of *Map* infection may occur in asymptomatic hosts that continue to shed viable bacteria in their faeces, leading to the infection of other healthy animals. Using multi-locus short sequence repeat (ML-SSR) analysis we previously reported that individual Johne’s positive dairy cattle from farms across the island of Newfoundland were infected by *Map* with multiple SSR-types simultaneously. The occurrence of multiple mixed genotype infections has the potential to change pathogen and disease dynamics as well as reduce the efficacy of treatments and vaccines. Therefore, we conducted whole genome sequencing (WGS) and single nucleotide polymorphism (SNP) analysis on a subset of these isolates for a more in-depth examination. We also implemented a PCR assay using two discriminatory SNPs and demonstrated the incidence of a mixed infection by three genotypically diverse *Map* isolates in a single animal. In addition, results show that WGS and SNP analysis can provide a better understanding of the relationship between *Map* isolates from individual and different animals. In the future such studies on the occurrence of mixed genotype infections could potentially lead to the identification of variable pathogenicity of different genotypes and allow for better tracking of *Map* isolates for epidemiological studies.

## Introduction

The genus *Mycobacterium* is comprised of acid-fast bacilli, some of which are pathogenic and cause severe disease in humans and animals. For example, *M. tuberculosis* and *M. leprae* are the causative agents of tuberculosis and leprosy in humans, respectively. In addition, *M. avium* and *M. intracellulare*, two well defined species from the *M. avium* complex (MAC), have been linked to several diseases in humans and animals including Johne’s disease (JD) in ruminants (Mijs et al., 2002). JD is a chronic wasting disease affecting cattle, sheep, goats and other ruminants caused by *M. avium* subspecies *paratuberculosis* (*Map)* (Harris & Barletta, 2001). Rabbit, deer and other wildlife have also been shown to be infected by *Map* (Waddell et al., 2016). While various species of wildlife can act as a reservoir of *Map* (Nugent et al., 2011), the clinical symptoms of JD have only been observed in ruminants (Koets, Eda & Sreevatsan, 2015), camelids (Ghosh et al., 2012) and rabbits/hare (Beard et al., 2001). The symptoms of JD in infected animals are highly similar to those of Crohn’s disease in humans, where the absorptive surface of the gut is reduced due to thickening of the intestinal wall and other factors (Ott, Wells & Wagner, 1999). It has even been suggested that *Map* may play a role in Crohn’s disease (Eckburg & Relman, 2007), a notion that is still under debate (Bosca-Watts et al., 2015).

*Map* infections lead to significant economic losses in the dairy and beef industries due to lower yields of milk, reduced slaughter value and premature culling of infected animals (Ott, Wells & Wagner, 1999). Major obstacles in mitigating losses include the lack of affordable treatments that are licensed for food animals (Hermon-Taylor & Bull, 2002) or an effective vaccine that can guarantee complete protection of uninfected animals (Knust et al., 2013). It has also been shown that the early stages of *Map* infection can occur in asymptomatic hosts that continue to shed viable bacteria in their faeces, leading to subsequent infection of other susceptible individuals (Whitlock et al., 2000). Therefore, there has been a lot of recent interest in understanding the dissemination of *Map* using source tracking and epidemiological studies (Ahlstrom et al., 2015; Ahlstrom et al., 2016; Leão et al., 2016; Bryant et al., 2016).

While classically defined co-infection refers to cases involving two or more different species of pathogens, it can also include instances where genotypically different strains of the same species of pathogen are involved (referred to as mixed genotype infection) (Cox, 2001). Mixed genotype infections caused by a single bacterial species appear to be quite common (Balmer & Tanner, 2011), but are often overlooked or missed (Read & Taylor, 2001). Therefore, studying JD transmission and dissemination could be further complicated by intra-host evolution of *Map* or by the co-infection of hosts by multiple genetically divergent *Map* strains. Whole genome sequencing (WGS) offers a rapid and more precise tool for investigating infectious disease epidemiology compared to the traditionally used methods (Eyre et al., 2012; Eyre et al., 2013). However, WGS is often performed on a single isolate/colony from an individual due to time and financial constraints. If a mixed genotype infection is present, the analysis of a single isolate can completely miss identical, similar or divergent strains infecting the donor and recipient, leading to inaccurate conclusions about transmission (Van den Berg et al., 2005). Therefore, it has been suggested that epidemiological studies require the analysis of multiple isolates from an individual to accurately trace transmission (Döpfer et al., 2008).

Many bacterial infection outbreak and surveillance studies have employed molecular techniques, including WGS, for detecting varying degrees of mixed genotype infections for other bacterial pathogens (Wang et al., 1999; St Sauver et al., 2000; Aranda, Fagundes-Neto & Scaletsky, 2004; Cespedes et al., 2005; Ugolotti et al., 2016). There have also been numerous WGS studies that have examined the genetic diversity of *Map* from dairy animals (Ahlstrom et al., 2015; Bryant et al., 2016; Ahlstrom et al., 2016; Yue et al., 2016; Leão et al., 2016), but none which have analyzed or addressed mixed *Map* genotype infections using multiple isolates from a single animal. We recently reported that *Map* with different short sequence repeat (SSR) types could be isolated from individual dairy animals from the island of Newfoundland (Podder et al., 2015). Here we use WGS, single nucleotide polymorphism (SNP) calling and phylogenetics to analyze six *Map* isolates, three of which were isolated from a single animal. Our results demonstrate the occurrence of co-infection by genetically distinct *Map* isolates in Newfoundland dairy cattle.

## Materials and Methods

### Ethics statement

The study was approved by the Institutional Animal Care Committee (IACC, Memorial University of Newfoundland) as an “A” rated protocol (Number: 15-01-KT) because the samples used in the study were obtained from routine veterinary diagnostic submissions unrelated to this research. The report describes molecular and WGS analysis on previously isolated bacteria and did not directly involve any animals.

### WGS and molecular typing of isolates

Sample collection, isolation/culture of *Map* isolates, DNA extraction and subsequent manipulations were carried out as described previously (Podder et al., 2015). Six genetically distinct *Map* isolates were selected based on SSR profiles (Podder et al., 2015) and DNA was sent for WGS to The Centre for Applied Genomics (The Hospital for Sick Children, Toronto, Canada). Nextera XT libraries were prepared and sequence data was gathered using the Illumina HiSeq platform (Illumina, Inc. USA) with an average read depth of 1,000× coverage. *De novo* assembly of the six genomes was carried out using the A5 pipeline (Tritt et al., 2012), yielding 98, 90, 94, 90, 97 and 90 contigs for *Map* isolates 89C, 93B, 95A, 95B, 95E and 96E, respectively. Annotations were performed using the National Center for Biotechnology Information (NCBI) Prokaryotic Genome Annotation Pipeline (PGAP) and the Rapid Annotations based on Subsystem Technology (RAST) servers (Aziz et al., 2008; Brettin et al., 2015; Overbeek et al., 2014), which were analyzed using the Geneious R8 software package (Biomatters Ltd., Auckland, New Zealand). The Whole Genome Shotgun sequences for the six *Map* isolates have been deposited in GenBank under the BioSample accession numbers LGRY00000000 (89C), LGRZ00000000 (93B), LGSA00000000 (95A), LGSB00000000 (95B), LGSC00000000 (95E) and LGSD00000000 (96E). Using a previously established protocol, raw reads were analyzed at the SNP level for comparison against the reference *Map* K10 genome (Table S1) and previously sequenced Canadian isolates representative of different phylogenetic clades present in the country (Ahlstrom et al., 2016). The isolate with the highest depth of coverage from each Canadian clade containing more than one isolate was selected for comparison. An alignment of concatenated SNPs was used to produce a maximum likelihood phylogenetic dendrogram using PhyML (Guindon & Gascuel, 2003) with the TPM1uf nucleotide substitution model as determined by jModelTest (Darriba et al., 2012), and 100 bootstrap pseudo-replications to evaluate node support.

A confirmatory PCR assay was designed to amplify a 110 bp DNA fragment using the primer pair F: CTCCTTTCGGCCGCTGTA and R: AGCCCATTCGCTCCGTAT. Two differentiating SNPs identified in the WGS analysis (SNP171 and SNP172), which are in close physical proximity to one another in the genome, were targeted with this single PCR and Sanger sequencing assay using the same set of primers described above (Fig. S1). The assay was first tested using chromosomal DNA from each isolate as a template. Total DNA was extracted from four primary liquid cultures (89, 93, 95 and 96, the same ones that led to the six *Map* isolates) for subsequent PCR/sequence analysis using the MagMax Total Nucleic Acid Isolation Kit (Thermo Fisher Scientific, Waltham, MA, USA). A no-template DNA negative control was included in the assay, however no positive control was utilized because the amplicons were subsequently sequenced to determine if SNPs were present or not. This assay was utilized to confirm that specific SNPs associated with each isolate could be detected in the original sample and that sample 95 actually contained DNA from the three individual *Map* isolates used in the WGS analysis.

## Results and Discussion

We recently reported that on more than one occasion, *Map* with different SSR types could be isolated from single animals from dairy farms from Newfoundland (NL), Canada (Podder et al., 2015), suggesting mixed genotype infections. Our previous SSR study only analyzed four genetic loci/repeats to examine genetic diversity. These repetitive loci are known to be unstable (Van Belkum et al., 1998; Kasnitz et al., 2013); therefore, whether the different SSR types indeed represented distinct strains was yet to be confirmed. We used WGS, SNP calling and phylogenetics to analyze six NL *Map* isolates with different SSR-types (Podder et al., 2015) with the purpose examining their genetic relatedness/diversity at a higher resolution. The NL *Map* isolates 95A, 95B and 95E all originated from the same animal, whereas the isolates 89C, 93B and 96E came from different animals located on separate farms (Podder et al., 2015). The use of NCBI’s PGAP and the RAST servers allowed for annotation of the six NL *Map* genome sequences and their comparison with the revised *Map* K10 reference sequence (Wynne et al., 2010) (Table 1).

**Table 1:** Characteristics of genome sequences of *Map* isolates from Newfoundland and their comparison with the reference K10 strain. The table shows the sizes of the respective genomes (in Mb or megabases) and the numbers of SNPs that were identified in the current study. Details regarding sequencing and analysis are provided in the text of the manuscript.

Isolate/Strain^a^							
Characteristics	K10^b^	89C	93B	95A^c^	95B^c^	95E^c^	96E
Genome size (Mb)	4.829	4.777	4.768	4.776	4.772	4.774	4.771
SNPs relative to K10	NA^d^	196	74	94	74	84	72
Strain-specific (unique) SNPs ^e^	NA^d^	157	4	64	3	46	1

**Notes.**

a The WGS data for the NL *Map* isolates represent genomes at the contiguous sequence level, which are not closed or completed.

b The revised (Wynne et al., 2010) genome sequence of the K10 strain in the public database was used for comparison.

c The three isolates were derived from a single animal whereas all others came from separate animals from different farms. For the Newfoundland isolates, the first number refers to the identity of the animal sampled followed by a letter assigned to a specific isolated *Map* colony used in the subsequent analysis (Podder et al., 2015).

d NA, not applicable as the sequence was used for identifying variant SNPs (single nucleotide polymorphisms) in the other isolates.

e These SNPs were only present in the one isolate each analyzed in the current study.

WGS results showed that all of the isolates have a highly similar genome size of approximately 4.77 Mb and are smaller than the ∼4.83 Mb K10 reference genome, which can be explained as our sequences do not represent fully closed genomes (Table 1). The notable similarity between our isolates and K10 was not surprising as *Map* is known to exhibit low genetic heterogeneity (Collins & De Lisle, 1986; Möbius et al., 2015). SNP analysis showed that *Map* 89C has nearly 2.0–2.6 times the number of total SNPs relative to K10 when compared to the other NL *Map* isolates included in the study (Table 1). From the total SNPs we calculated the number of SNPs that were unique to each of the six NL *Map* isolates within our dataset. It was observed that 89C has by far the largest number of unique SNPs (*n* = 157), whereas isolates 93B, 95B and 96E range from only 1–4 (Table 1). Of particular interest are isolates 95A, 95B and 95E from the same animal. The pairwise SNP differences between these isolates ranged from 115 to 125 (Table S2). This was considered to be a significant finding because it has been estimated that *Map* accumulates SNPs at a slower rate than *M. tuberculosis* (0.3 SNPs per genome per year) (Bryant et al., 2013; Bryant et al., 2016). As such, the molecular clock of *Map* is too slow to account for within-host evolution based on the numbers of unique SNPs observed in isolates 95A, 95B and 95E, supporting that a mixed genotype infection by genetically distinct strains had occurred.

**Figure 1:**
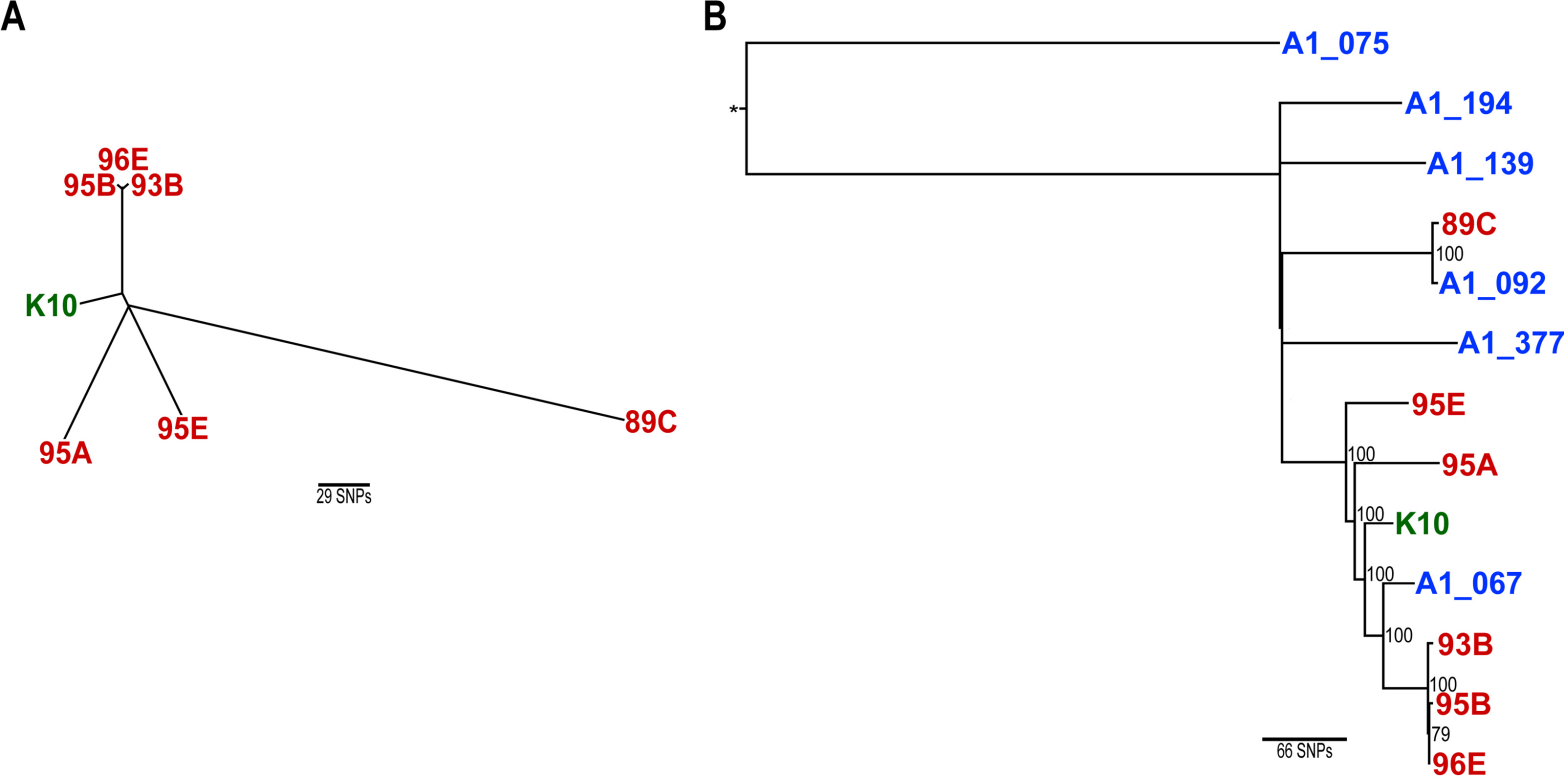
Maximum likelihood phylogenetic trees based on concatenated SNPs using the TPM1uf nucleotide substitution model (Ahlstrom et al., 2015). (A) Unrooted phylogenetic tree of the Newfoundland *Map* isolates and the K10 reference strain. (B) Phylogenetic rooted tree including the Newfoundland isolates and representative Canadian Map isolates identified in a previous study (Ahlstrom et al., 2016). The tree was rooted using a divergent *Map* isolate (represented by the star) unrelated to the current study and is denoted by an asterisk. Bootstrap values with branch support equal to or greater than 70% are displayed in black. (A and B) Newfoundland isolates are displayed in red, the K10 reference strain is displayed in green and representative Canadian *Map* isolates are displayed in blue.

To visualize the genetic relatedness/divergence between the six *Map* isolates from Newfoundland and the reference K10 strain, phylogenetic analysis was performed using a concatenated sequence of SNPs from each isolate (Fig. 1A). Results show that the three isolates from the single animal (95A, B and E) do not cluster together in a phylogenetic tree, which further supports a mixed genotype infection. While 95A, B and E are genetically distinct from one another, 95B clustered with 93B and 96E. This would seem to suggest that 95A, 95B and 95E are co-infecting as genetically divergent strains, and that 95B may be epidemiologically related to 93B and 96E isolated from dairy cattle on two different farms.

Similar phylogenetic analysis was also conducted using SNP profiles from divergent *Map* subtypes previously described from Canada, one of which encompasses 86% of all Canadian isolates (Ahlstrom et al., 2016). Figure 1B shows the genetic relationship between the NL *Map* isolates and also places them among six isolates A1_075, A1_194, A1_139, A1_377, A1_092 and A1_067 which belong to the Canadian *Map* clades A, C, D, E, F and H, respectively (Ahlstrom et al., 2016). As expected, 95A, 95B and 95E do not cluster together, but isolates 93B, 95B and 96E do cluster together despite the fact that each one originated from a different farm, suggesting a recent transmission event occurred between these farms, either directly or indirectly. The movement of cattle within and between provinces in the Canadian dairy industry is extensive and is thought to be a major contributor to herd level *Map* transmission (Ahlstrom et al., 2016). Furthermore, over the last decade more than 200,000 cattle have been imported from the United States of America (Canadian Dairy Information Centre (CDIC), 2013). It should therefore not be surprising that genetic relatedness between geographically distant isolates is observed. Further information regarding a link between these farms, such as cattle purchases, could elucidate how such genetically related *Map* isolates were geographically dispersed.

In addition to the WGS and SNP analysis, a confirmatory PCR and sequencing assay was developed to detect unique differentiating SNPs (SNP171 and SNP172) found in the individual isolates (Table 2). SNP171 and SNP172 are located at positions 2,316,042 and 2,316,081 in the revised K10 genome (Wynne et al., 2010) respectively, within a non-coding region between the genes *mapk_2034* (PPE-repeat protein) and *mapk_2035* (Ren71). The assay was tested using chromosomal DNA isolated from axenic *Map* cultures as a template (Table 2) and was then applied to DNA extracted from the primary cultures inoculated with fecal samples from respective infected animals (Table 2). These primary cultures were the ones that were later plated onto solid agar media to pick individual isolated colonies for establishing axenic cultures for WGS analysis (Podder et al., 2015). The purpose of the PCR/sequencing assay was to ensure that the respective SNP variants found in the individual isolates could also be detected in the original primary cultures. For instance, the 95A, 95B and 95E isolates have T/T/G as SNP172 and A/G/A at SNP171, respectively (Table 2); thus, these alleles should also be detected in DNA extracts from the primary mixed culture (Table 2). In such cases, more than one nucleotide/allele was identified at both loci in DNA extracts from primary cultures (Table 2), which appeared as “doublet” peaks during sequencing. The results confirmed that primary culture 95 contained DNA associated with the 95A, 95B and 95E isolates and that a mixed genotype infection occurred.

**Table 2:** Analysis of two discriminatory SNPs in chromosomal DNA isolated from individual *Map* isolates and DNA extracted from non-axenic primary cultures. The identities of the nucleotides were determined using the PCR and sequencing assay that is described under the materials and methods section. The primary cultures were the animal derived mixed cultures that led to the isolation of the individual Map isolates for subsequent analysis.

Isolate/Sample^a^	SNP172 isolate (primary)^b^	SNP171 isolate (primary)^b^
K10	T	T
89C/89	ND^c^ (T)	G (G)
93B/93	C (T)	G (G)
95A/95	T (T/G)^d^	A (G/A)^d^
95B/95	T (T/G)^d^	G (G/A)^d^
95E/95	G (T/G)^d^	A (G/A)^d^
96E/96	T (T)	G (G)

**Notes.**

a Identity of axenic isolate or primary culture used to extract template DNA used in the analysis. The assigned number refers to the identity of the animal from which the primary sample was derived followed by a letter assigned to a specific isolated *Map* colony used in the subsequent analysis (Podder et al., 2015).

b After PCR amplification the identity of the nucleotide associated with the SNP in chromosomal DNA from each isolate (underlined) and in total DNA from primary cultures (shown in parenthesis) was determined using Sanger sequencing.

c ND, none detected. A variant SNP was not detected at this location in the isolate when compared to the reference K10 strain.

d In some cases more than one nucleotide was detected during analysis of total DNA from primary cultures, which corresponds with the SNPs identified in the separate isolates.

There is evidence for mixed genotype infections by mycobacteria, including other subspecies of *M. avium*. One such example is a study showing that HIV-positive inmates in a Spanish prison were at risk of exogenous reinfection with multiple strains of *M. tuberculosis*, some of which had different drug susceptibilities (Chaves et al., 1999). In a separate study (Theisen et al., 1995), infection by two strains of *M. tuberculosis* with different drug susceptibilities was also reported in an immunocompetent patient. Additionally, multiple strains of *M. tuberculosis* have been identified in single sputum specimens from patients with active tuberculosis (Warren et al., 2004). Mixed genotype infections by *M. avium* subspecies *avium* have also been observed. It was found that certain AIDS patients were sometimes infected with multiple strains of *M. avium* subspecies *avium* (Arbeit et al., 1993). Similarly, a mixed *M. avium* subspecies *avium* infection in chickens was also reported following examination of DNA isolated from fecal samples (Shitaye et al., 2008). Recently, a mixed infection by *M. bovis* was reported in cattle diagnosed with bovine tuberculosis and microevolution of the isolates was also characterized (Navarro et al., 2015; Navarro et al., 2016). However, it should be noted that all of the studies mentioned above used conventional molecular typing methods and not WGS analysis.

Overall, our results on the six Newfoundland *Map* isolates suggest that co-infection of a single dairy cow with at least three different isolates (95A, 95B and 95E) occurred. The biological and clinical significance of understanding mixed genotype infections should not be understated (Cox, 2001). Distinguishing individual strains playing a role during an infection can be problematic, making full understanding of the evolution of pathogens and disease progression difficult. Lacking this understanding, the predicted outcome of mixed genotype infections on disease dynamics and pathogenesis, as well as on treatments and vaccines is limited (Balmer & Tanner, 2011; Navarro et al., 2015). The decision to analyze/sequence a single colony from each sample/specimen is typically made because of financial and logistical considerations in clinical laboratories or in large-scale transmission studies (Van den Berg et al., 2005; Eyre et al., 2013), but the value of analyzing multiple isolates is clear. In the future, it will be interesting to conduct an extensive study on the prevalence of *Map* mixed genotype infections, which we predict are frequent (Podder et al., 2015). Our results also demonstrate the application of WGS and SNP analysis as a high resolution tool for analyzing mixed genotype infections and for studying intra-host evolution of a pathogen during the process.

##  Supplemental Information

10.7717/peerj.2793/supp-1 Figure S1 Nucleotide sequence of the genetic locus including the SNPs targeted in the PCR assay used to discriminate between the different *Map* isolates described in the study The DNA sequence of the genetic locus targeted in the PCR assay that was used to discriminate between the different *Map* isolates is shown. The sequence corresponds to that present in the * Map* K-10 reference strain (positive control) and the two SNPs (SNP171 and SNP172) used in the analysis are marked. The arrows indicate the sequences of the forward (F) and reverse (R) primers used for PCR amplification and subsequent DNA sequence analysis.

10.7717/peerj.2793/supp-2 Table S1 List of different SNPs identified in the NL *Map* isolates when compared to the reference K-10 strain Details regarding SNP identification and analysis are described in the text of the manuscript.

10.7717/peerj.2793/supp-3 Table S2 Distance matrix based on pairwise SNP differences between NL *Map* isolates, representative Canadian isolates and the reference K-10 strain Details regarding SNP identification and analysis are described in the text of the manuscript.

## Additional Information and Declarations

### DNA Deposition

The following information was supplied regarding the deposition of DNA sequences: The Whole Genome Shotgun sequences for the six Map isolates have been deposited in GenBank under the accession numbers LGRY00000000 (89C), LGRZ00000000 (93B), LGSA00000000 (95A), LGSB00000000 (95B), LGSC00000000 (95E) and LGSD00000000 (96E).
