# Supplementary material for: Examination of Mycobacterium avium subspecies paratuberculosis mixed genotype infections in dairy animals using a whole genome sequencing approach
Source: PeerJ. 2016 Dec 14;4:e2793. doi: 10.7717/peerj.2793 (PMC5160890; doi:10.7717/peerj.2793)
Supplement: Figure S1 — The DNA sequence of the genetic locus targeted in the PCR assay that was used to discriminate between the different Map isolates is shown. The sequence corresponds to that present in the Map K-10 reference strain (positive control) and the two SNPs (SNP171 and SNP172) used in the analysis are marked. The arrows indicate the sequences of the forward (F) and reverse (R) primers used for PCR amplification and subsequent DNA sequence analysis. [file peerj-04-2793-s001.pdf]

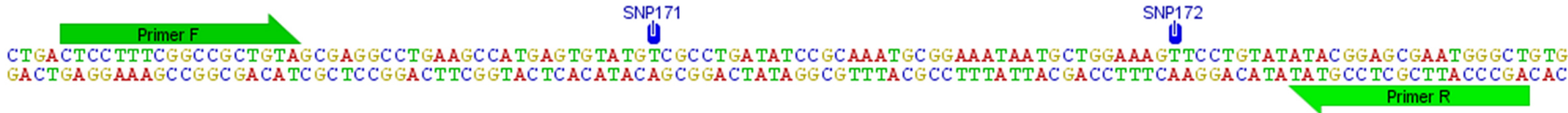
SNP171
SNP172
Primer F
CTGACTCCTTTCGGCCGCTGTAGCGAGGCCTGAAGCCATGAGTGTATGTCGCCTGATATCCGCAAATGCGGAAATAATGCTGGAAAGTTCCTGTATATACGGAGCGAATGGGCTGTG
GACTGAGGAAAGCCGGCGACATCGCTCCGGACTTCGGTACTCACATACAGCGGACTATAGGCGTTTACGCCTTTATTACGACCTTTCAAGGACATATATGCCTCGCTTACCCGACAC
Primer R
