# Supplementary material for: Examination of Mycobacterium avium subspecies paratuberculosis mixed genotype infections in dairy animals using a whole genome sequencing approach
Source: PeerJ. 2016 Dec 14;4:e2793. doi: 10.7717/peerj.2793 (PMC5160890; doi:10.7717/peerj.2793)
Supplement: Table S1 — Details regarding SNP identification and analysis are described in the text of the manuscript. [file peerj-04-2793-s002.docx]

**Supplementary Table 1.** List of different SNPs identified in the NL *Map* isolates when compared to the reference K-10 strain.

| **Position ^a^** | **REF ^b^** | **ALT ^c^** | **K89C** | **K93B** | **K95A** | **K95B** | **K95E** | **K96E** |
| --- | --- | --- | --- | --- | --- | --- | --- | --- |
| 752 | T | C | ALT | ALT | ALT | ALT | ALT | ALT |
| 19101 | G | C | ALT | REF | REF | REF | REF | REF |
| 30901 | A | C | REF | REF | REF | REF | ALT | REF |
| 49240 | C | T | REF | REF | ALT | REF | REF | REF |
| 56276 | T | C | REF | ALT | REF | ALT | REF | ALT |
| 71014 | A | G | ALT | REF | REF | REF | REF | REF |
| 73211 | T | C | ALT | ALT | ALT | ALT | ALT | ALT |
| 76286 | G | T | REF | REF | REF | REF | ALT | REF |
| 87442 | A | G | ALT | REF | REF | REF | REF | REF |
| 91027 | C | T | ALT | REF | REF | REF | REF | REF |
| 91310 | A | G | ALT | ALT | ALT | ALT | ALT | ALT |
| 99927 | A | G | ALT | REF | REF | REF | REF | REF |
| 106690 | G | A | REF | REF | REF | ALT | REF | ALT |
| 119654 | G | A | ALT | REF | REF | REF | REF | REF |
| 122160 | C | G | REF | ALT | REF | ALT | REF | ALT |
| 142835 | G | C | ALT | REF | REF | REF | REF | REF |
| 143639 | T | C | REF | ALT | REF | ALT | REF | ALT |
| 150857 | G | C | ALT | REF | REF | REF | REF | REF |
| 155252 | C | T | ALT | REF | REF | REF | REF | REF |
| 163876 | A | G | ALT | REF | REF | REF | REF | REF |
| 167794 | C | A | REF | REF | REF | REF | ALT | REF |
| 173146 | C | T | REF | REF | REF | REF | ALT | REF |
| 186552 | G | A | REF | ALT | REF | ALT | REF | ALT |
| 188304 | C | T | ALT | REF | REF | REF | REF | REF |
| 189413 | A | G | ALT | REF | REF | REF | REF | REF |
| 222613 | C | T | ALT | REF | REF | REF | REF | REF |
| 223602 | A | G | ALT | REF | REF | REF | REF | REF |
| 232136 | C | T | REF | ALT | REF | ALT | REF | ALT |
| 256902 | C | T | REF | REF | REF | REF | ALT | REF |
| 263781 | G | T | ALT | REF | REF | REF | REF | REF |
| 274291 | C | G | REF | REF | ALT | REF | REF | REF |
| 320885 | C | G | REF | REF | REF | REF | ALT | REF |
| 325001 | C | G | ALT | REF | REF | REF | REF | REF |
| 337197 | C | T | REF | REF | ALT | REF | REF | REF |
| 339762 | C | T | REF | REF | ALT | REF | REF | REF |
| 364818 | A | G | REF | ALT | REF | ALT | REF | ALT |
| 391388 | C | T | ALT | REF | REF | REF | REF | REF |
| 426119 | A | G | REF | ALT | REF | ALT | REF | ALT |
| 454430 | T | G | ALT | ALT | ALT | ALT | ALT | ALT |
| 476636 | C | T | REF | REF | REF | REF | ALT | REF |
| 497165 | G | A | REF | REF | ALT | REF | REF | REF |
| 498056 | C | G | REF | REF | ALT | REF | REF | REF |
| 508728 | C | T | ALT | REF | REF | REF | REF | REF |
| 558559 | G | A | ALT | REF | REF | REF | REF | REF |
| 595788 | T | C | ALT | REF | REF | REF | REF | REF |
| 599616 | C | T | REF | REF | REF | REF | ALT | REF |
| 644387 | A | G | REF | REF | ALT | REF | REF | REF |
| 660570 | C | T | REF | REF | REF | REF | ALT | REF |
| 667295 | T | G | REF | REF | REF | REF | ALT | REF |
| 693293 | G | C | ALT | ALT | ALT | ALT | ALT | ALT |
| 693483 | G | T | REF | ALT | REF | ALT | REF | ALT |
| 723001 | A | G | ALT | REF | REF | REF | REF | REF |
| 746361 | C | A | ALT | REF | REF | REF | REF | REF |
| 757437 | T | G | REF | ALT | REF | ALT | REF | ALT |
| 784104 | T | C | REF | ALT | REF | ALT | REF | ALT |
| 791303 | A | G | REF | REF | ALT | REF | REF | REF |
| 792142 | C | T | REF | REF | ALT | REF | REF | REF |
| 860934 | A | C | REF | REF | REF | REF | ALT | REF |
| 867569 | C | T | REF | REF | REF | REF | ALT | REF |
| 870453 | C | T | ALT | REF | REF | REF | REF | REF |
| 874128 | C | T | REF | ALT | REF | ALT | REF | ALT |
| 884216 | A | C | ALT | REF | REF | REF | REF | REF |
| 892773 | A | G | ALT | REF | REF | REF | REF | REF |
| 892816 | G | A | ALT | REF | REF | REF | REF | REF |
| 898531 | C | G | ALT | REF | REF | REF | ALT | REF |
| 902488 | C | T | REF | ALT | REF | ALT | REF | ALT |
| 922205 | G | A | REF | REF | REF | REF | ALT | REF |
| 924886 | C | T | REF | ALT | REF | ALT | REF | ALT |
| 927445 | C | T | ALT | REF | REF | REF | REF | REF |
| 932710 | C | G | REF | ALT | REF | ALT | REF | ALT |
| 934275 | G | C | REF | REF | ALT | REF | REF | REF |
| 943770 | G | T | REF | REF | REF | REF | ALT | REF |
| 946562 | C | T | REF | REF | REF | REF | ALT | REF |
| 960250 | G | C | ALT | REF | REF | REF | REF | REF |
| 1004876 | T | C | REF | ALT | REF | ALT | REF | ALT |
| 1008207 | C | T | REF | REF | ALT | REF | REF | REF |
| 1046642 | A | G | REF | REF | ALT | REF | REF | REF |
| 1066097 | C | T | REF | REF | ALT | REF | REF | REF |
| 1085337 | A | C | ALT | REF | REF | REF | REF | REF |
| 1104359 | G | C | REF | ALT | REF | REF | REF | REF |
| 1120495 | C | T | ALT | REF | REF | REF | REF | REF |
| 1136742 | G | A | REF | REF | ALT | REF | REF | REF |
| 1147674 | C | G | ALT | REF | REF | REF | REF | REF |
| 1161915 | C | T | REF | REF | REF | REF | ALT | REF |
| 1169976 | A | C | ALT | ALT | ALT | ALT | ALT | ALT |
| 1185645 | T | A | ALT | REF | REF | REF | REF | REF |
| 1193314 | T | C | ALT | REF | REF | REF | REF | REF |
| 1204735 | T | C | ALT | ALT | ALT | ALT | ALT | ALT |
| 1212096 | G | A | ALT | REF | REF | REF | REF | REF |
| 1218204 | G | C | ALT | REF | ALT | REF | ALT | REF |
| 1220862 | C | T | REF | REF | REF | REF | ALT | REF |
| 1224368 | C | T | ALT | REF | REF | REF | REF | REF |
| 1227540 | A | G | ALT | REF | REF | REF | REF | REF |
| 1230312 | G | A | REF | REF | ALT | REF | REF | REF |
| 1233762 | G | A | REF | ALT | REF | ALT | REF | ALT |
| 1233871 | T | G | ALT | REF | REF | REF | REF | REF |
| 1255429 | C | T | REF | ALT | REF | ALT | REF | ALT |
| 1263872 | G | A | ALT | REF | REF | REF | REF | REF |
| 1287459 | C | T | ALT | REF | REF | REF | REF | REF |
| 1297534 | T | C | ALT | REF | REF | REF | REF | REF |
| 1306289 | T | C | ALT | REF | REF | REF | REF | REF |
| 1361980 | T | G | REF | REF | REF | REF | ALT | REF |
| 1362204 | C | T | ALT | REF | REF | REF | REF | REF |
| 1363662 | A | C | REF | ALT | REF | ALT | REF | ALT |
| 1378948 | A | C | ALT | REF | ALT | REF | ALT | REF |
| 1379097 | C | T | ALT | REF | REF | REF | REF | REF |
| 1393230 | T | C | ALT | REF | REF | REF | REF | REF |
| 1403374 | C | T | ALT | REF | REF | REF | REF | REF |
| 1424433 | T | G | REF | REF | REF | REF | ALT | REF |
| 1429205 | C | G | REF | REF | REF | REF | ALT | REF |
| 1469245 | T | G | REF | REF | ALT | REF | REF | REF |
| 1485890 | C | T | REF | REF | ALT | REF | REF | REF |
| 1486554 | A | G | ALT | REF | REF | REF | REF | REF |
| 1491382 | G | C | ALT | REF | REF | REF | REF | REF |
| 1498881 | G | A | REF | ALT | REF | REF | REF | REF |
| 1500553 | C | T | REF | ALT | REF | ALT | REF | ALT |
| 1506314 | T | C | ALT | REF | REF | REF | REF | REF |
| 1508495 | C | T | REF | REF | REF | REF | ALT | REF |
| 1553750 | G | A | REF | REF | REF | ALT | REF | REF |
| 1561113 | T | C | REF | REF | ALT | REF | REF | REF |
| 1607571 | A | G | REF | ALT | REF | ALT | REF | ALT |
| 1621714 | G | A | REF | REF | REF | REF | ALT | REF |
| 1629902 | G | A | REF | REF | ALT | REF | REF | REF |
| 1637809 | C | A | REF | REF | ALT | REF | REF | REF |
| 1666010 | C | A | ALT | REF | REF | REF | REF | REF |
| 1673701 | C | G | REF | ALT | REF | ALT | REF | ALT |
| 1686154 | T | C | ALT | REF | REF | REF | REF | REF |
| 1693832 | T | C | ALT | REF | REF | REF | REF | REF |
| 1699767 | C | G | ALT | REF | REF | REF | REF | REF |
| 1700471 | C | T | ALT | REF | REF | REF | REF | REF |
| 1703429 | G | A | ALT | REF | REF | REF | REF | REF |
| 1728119 | C | T | ALT | REF | REF | REF | REF | REF |
| 1729551 | C | G | ALT | REF | REF | REF | REF | REF |
| 1744979 | C | T | REF | REF | ALT | REF | REF | REF |
| 1753759 | C | T | ALT | REF | REF | REF | REF | REF |
| 1754956 | C | G | ALT | REF | REF | REF | REF | REF |
| 1796038 | C | A | ALT | REF | REF | REF | REF | REF |
| 1800207 | G | T | ALT | REF | REF | REF | REF | REF |
| 1819487 | G | A | REF | REF | ALT | REF | REF | REF |
| 1910469 | T | C | ALT | REF | ALT | REF | ALT | REF |
| 1912666 | G | C | ALT | REF | REF | REF | REF | REF |
| 1956235 | T | C | ALT | REF | REF | REF | REF | REF |
| 1964085 | A | G | ALT | REF | REF | REF | REF | REF |
| 1982725 | A | G | REF | ALT | REF | ALT | REF | ALT |
| 1997125 | C | T | REF | REF | ALT | REF | REF | REF |
| 1998323 | C | T | REF | ALT | REF | ALT | REF | ALT |
| 2012374 | C | T | ALT | REF | REF | REF | REF | REF |
| 2018876 | G | A | REF | REF | ALT | REF | REF | REF |
| 2020086 | C | T | ALT | REF | REF | REF | REF | REF |
| 2041445 | T | C | ALT | ALT | ALT | ALT | ALT | ALT |
| 2042770 | T | C | REF | REF | ALT | REF | REF | REF |
| 2054875 | T | C | ALT | REF | REF | REF | REF | REF |
| 2059651 | T | C | ALT | REF | REF | REF | REF | REF |
| 2083177 | T | C | ALT | REF | REF | REF | REF | REF |
| 2103426 | G | A | REF | REF | REF | REF | ALT | REF |
| 2110866 | C | T | ALT | REF | REF | REF | REF | REF |
| 2119762 | G | A | ALT | REF | REF | REF | REF | REF |
| 2150323 | A | G | REF | REF | ALT | REF | REF | REF |
| 2161992 | C | A | REF | ALT | REF | ALT | REF | ALT |
| 2172538 | C | G | ALT | REF | REF | REF | REF | REF |
| 2189617 | A | C | REF | REF | REF | REF | ALT | REF |
| 2191540 | C | T | ALT | REF | REF | REF | REF | REF |
| 2193870 | C | T | REF | REF | ALT | REF | REF | REF |
| 2214445 | C | T | ALT | REF | REF | REF | REF | REF |
| 2221871 | G | A | ALT | REF | REF | REF | REF | REF |
| 2255722 | T | C | REF | REF | REF | REF | ALT | REF |
| 2260595 | G | A | ALT | REF | REF | REF | REF | REF |
| 2262766 | G | A | ALT | REF | REF | REF | REF | REF |
| 2283471 | C | A | REF | REF | ALT | REF | REF | REF |
| 2299755 | C | A | ALT | REF | REF | REF | REF | REF |
| 2316042 | T | C | ALT | ALT | REF | ALT | REF | ALT |
| 2316081 | T | G | REF | REF | REF | REF | ALT | REF |
| 2317606 | C | T | REF | ALT | REF | ALT | REF | ALT |
| 2318764 | G | T | ALT | REF | REF | REF | REF | REF |
| 2320162 | G | A | REF | ALT | REF | REF | REF | REF |
| 2327379 | A | G | ALT | REF | REF | REF | ALT | REF |
| 2338670 | A | G | ALT | REF | REF | REF | REF | REF |
| 2345668 | A | G | ALT | REF | REF | REF | REF | REF |
| 2354248 | A | G | ALT | REF | REF | REF | REF | REF |
| 2356930 | A | G | REF | ALT | REF | ALT | REF | ALT |
| 2392848 | G | A | ALT | REF | REF | REF | REF | REF |
| 2418382 | A | G | ALT | REF | REF | REF | REF | REF |
| 2420747 | G | A | ALT | REF | REF | REF | REF | REF |
| 2431979 | G | A | ALT | REF | REF | REF | REF | REF |
| 2444270 | G | C | ALT | REF | REF | REF | REF | REF |
| 2491806 | C | A | ALT | REF | REF | REF | REF | REF |
| 2495259 | A | G | REF | REF | ALT | REF | REF | REF |
| 2498909 | A | G | REF | REF | ALT | REF | REF | REF |
| 2500615 | G | A | REF | REF | ALT | REF | REF | REF |
| 2503656 | G | A | ALT | REF | REF | REF | REF | REF |
| 2536677 | G | A | REF | REF | REF | REF | ALT | REF |
| 2542162 | A | G | ALT | REF | REF | REF | REF | REF |
| 2543626 | G | A | ALT | REF | REF | REF | REF | REF |
| 2558788 | A | G | ALT | REF | REF | REF | REF | REF |
| 2575293 | A | G | ALT | ALT | ALT | ALT | ALT | ALT |
| 2586747 | T | A | ALT | ALT | ALT | ALT | ALT | ALT |
| 2609794 | A | G | ALT | ALT | ALT | ALT | ALT | ALT |
| 2618176 | G | A | REF | REF | ALT | REF | REF | REF |
| 2633515 | A | G | ALT | REF | REF | REF | REF | REF |
| 2635429 | T | G | REF | REF | ALT | REF | REF | REF |
| 2635517 | A | G | ALT | REF | REF | REF | REF | REF |
| 2661355 | G | A | ALT | REF | REF | REF | REF | REF |
| 2679997 | G | A | ALT | REF | REF | REF | REF | REF |
| 2709520 | T | C | ALT | REF | REF | REF | REF | REF |
| 2719318 | T | C | ALT | REF | REF | REF | REF | REF |
| 2734345 | A | G | ALT | REF | REF | REF | REF | REF |
| 2737691 | T | C | REF | ALT | REF | ALT | REF | ALT |
| 2741506 | C | T | ALT | REF | REF | REF | REF | REF |
| 2758670 | C | A | REF | REF | REF | REF | ALT | REF |
| 2780298 | G | T | REF | REF | ALT | REF | REF | REF |
| 2797003 | G | A | REF | ALT | REF | ALT | REF | ALT |
| 2816060 | G | A | ALT | REF | REF | REF | REF | REF |
| 2830404 | C | T | ALT | REF | REF | REF | REF | REF |
| 2843276 | T | C | ALT | REF | REF | REF | REF | REF |
| 2851830 | C | T | REF | ALT | REF | ALT | REF | ALT |
| 2857982 | T | A | REF | ALT | REF | ALT | REF | ALT |
| 2860218 | A | G | ALT | REF | REF | REF | REF | REF |
| 2860465 | A | T | REF | REF | REF | REF | ALT | REF |
| 2867511 | A | G | REF | REF | ALT | REF | REF | REF |
| 2878537 | T | G | ALT | REF | REF | REF | ALT | REF |
| 2892775 | T | C | ALT | REF | REF | REF | REF | REF |
| 2907081 | C | A | REF | REF | REF | ALT | REF | REF |
| 2916607 | C | T | REF | REF | ALT | REF | REF | REF |
| 2924244 | G | A | REF | REF | ALT | REF | REF | REF |
| 2934514 | T | C | ALT | REF | REF | REF | REF | REF |
| 2941635 | G | T | REF | REF | REF | REF | ALT | REF |
| 2946017 | G | A | REF | ALT | REF | ALT | REF | ALT |
| 2946491 | C | G | ALT | REF | REF | REF | REF | REF |
| 2977162 | C | G | REF | REF | ALT | REF | REF | REF |
| 2979488 | C | T | ALT | REF | REF | REF | REF | REF |
| 2991209 | G | T | REF | ALT | REF | ALT | REF | ALT |
| 2995306 | G | T | REF | REF | REF | REF | ALT | REF |
| 3012675 | G | C | REF | REF | REF | REF | ALT | REF |
| 3020561 | A | G | ALT | REF | ALT | REF | ALT | REF |
| 3024417 | A | C | ALT | REF | REF | REF | REF | REF |
| 3029700 | C | T | ALT | ALT | ALT | ALT | ALT | ALT |
| 3068120 | C | T | REF | REF | ALT | REF | REF | REF |
| 3115307 | C | G | REF | REF | REF | REF | ALT | REF |
| 3131408 | G | A | REF | ALT | REF | ALT | REF | ALT |
| 3133871 | G | A | ALT | REF | ALT | REF | ALT | REF |
| 3135492 | G | A | REF | ALT | REF | REF | REF | REF |
| 3142300 | G | A | REF | REF | ALT | REF | REF | REF |
| 3156453 | T | C | REF | ALT | REF | ALT | REF | ALT |
| 3165441 | G | A | ALT | REF | REF | REF | REF | REF |
| 3188298 | A | T | ALT | REF | REF | REF | REF | REF |
| 3192835 | A | G | ALT | REF | REF | REF | REF | REF |
| 3200830 | G | A | REF | REF | ALT | REF | REF | REF |
| 3213938 | C | T | REF | ALT | REF | ALT | REF | ALT |
| 3251603 | T | G | REF | REF | REF | REF | ALT | REF |
| 3259329 | C | T | ALT | ALT | ALT | ALT | ALT | ALT |
| 3262486 | A | C | REF | REF | ALT | REF | REF | REF |
| 3278891 | A | T | ALT | ALT | ALT | ALT | ALT | ALT |
| 3304057 | G | A | REF | REF | REF | REF | ALT | REF |
| 3311838 | T | C | ALT | ALT | ALT | ALT | ALT | ALT |
| 3325161 | G | A | REF | REF | REF | REF | ALT | REF |
| 3335003 | G | C | REF | REF | ALT | REF | REF | REF |
| 3357387 | G | C | REF | ALT | REF | ALT | REF | ALT |
| 3358322 | G | C | REF | REF | ALT | REF | REF | REF |
| 3376200 | G | C | REF | REF | ALT | REF | REF | REF |
| 3385059 | T | G | ALT | REF | REF | REF | REF | REF |
| 3392116 | G | A | ALT | REF | REF | REF | REF | REF |
| 3394320 | T | A | ALT | REF | REF | REF | REF | REF |
| 3433030 | T | G | ALT | REF | REF | REF | REF | REF |
| 3448761 | C | T | REF | REF | ALT | REF | REF | REF |
| 3469583 | C | A | ALT | REF | REF | REF | REF | REF |
| 3471415 | G | A | REF | ALT | REF | ALT | REF | ALT |
| 3474857 | T | G | ALT | ALT | ALT | ALT | ALT | ALT |
| 3479753 | A | C | ALT | REF | REF | REF | REF | REF |
| 3488714 | C | G | ALT | REF | ALT | REF | ALT | REF |
| 3496761 | C | G | ALT | REF | REF | REF | REF | REF |
| 3498847 | A | G | ALT | REF | REF | REF | REF | REF |
| 3511639 | A | G | ALT | REF | REF | REF | REF | REF |
| 3525416 | T | G | ALT | REF | REF | REF | ALT | REF |
| 3530160 | A | G | ALT | REF | REF | REF | REF | REF |
| 3557148 | G | A | REF | REF | ALT | REF | REF | REF |
| 3561163 | A | G | REF | REF | ALT | REF | REF | REF |
| 3565035 | A | G | ALT | REF | REF | REF | REF | REF |
| 3566323 | T | C | ALT | REF | REF | REF | REF | REF |
| 3567305 | C | T | REF | REF | REF | REF | ALT | REF |
| 3579144 | A | G | ALT | REF | REF | REF | ALT | REF |
| 3592522 | T | C | ALT | REF | REF | REF | REF | REF |
| 3597190 | C | T | ALT | REF | REF | REF | REF | REF |
| 3613346 | C | G | REF | REF | ALT | REF | REF | REF |
| 3616982 | A | G | REF | ALT | REF | ALT | REF | ALT |
| 3625558 | C | T | ALT | REF | REF | REF | REF | REF |
| 3639796 | T | C | REF | REF | ALT | REF | REF | REF |
| 3675858 | G | A | REF | REF | ALT | REF | REF | REF |
| 3681359 | G | C | ALT | REF | REF | REF | ALT | REF |
| 3691538 | G | A | REF | REF | ALT | REF | REF | REF |
| 3713141 | A | T | REF | REF | REF | REF | ALT | REF |
| 3726660 | T | G | REF | REF | ALT | REF | REF | REF |
| 3730554 | A | C | REF | REF | ALT | REF | REF | REF |
| 3735166 | C | T | REF | REF | ALT | REF | REF | REF |
| 3738290 | A | G | ALT | REF | REF | REF | REF | REF |
| 3751994 | G | A | ALT | REF | REF | REF | REF | REF |
| 3761641 | T | G | REF | REF | REF | REF | ALT | REF |
| 3766032 | A | G | ALT | REF | REF | REF | REF | REF |
| 3768230 | G | A | ALT | REF | REF | REF | REF | REF |
| 3798450 | A | T | REF | ALT | REF | ALT | REF | ALT |
| 3804374 | G | C | ALT | REF | REF | REF | REF | REF |
| 3805044 | T | A | ALT | REF | REF | REF | REF | REF |
| 3806520 | C | A | ALT | REF | REF | REF | REF | REF |
| 3821820 | G | A | ALT | REF | REF | REF | REF | REF |
| 3827048 | T | C | ALT | REF | REF | REF | REF | REF |
| 3836246 | A | G | ALT | REF | REF | REF | REF | REF |
| 3845927 | T | G | REF | ALT | REF | ALT | REF | ALT |
| 3872744 | A | G | ALT | REF | REF | REF | ALT | REF |
| 3888659 | A | C | ALT | REF | ALT | REF | ALT | REF |
| 3899230 | G | A | REF | ALT | REF | ALT | REF | ALT |
| 3903248 | A | T | REF | ALT | REF | ALT | REF | ALT |
| 3906051 | A | G | REF | ALT | REF | ALT | REF | ALT |
| 3907440 | G | A | REF | REF | REF | REF | ALT | REF |
| 3910684 | A | G | ALT | REF | REF | REF | REF | REF |
| 3920369 | A | G | ALT | REF | REF | REF | REF | REF |
| 3953923 | G | C | REF | REF | REF | ALT | REF | REF |
| 3971919 | T | C | REF | REF | ALT | REF | REF | REF |
| 3979593 | C | G | REF | REF | ALT | REF | REF | REF |
| 3993334 | C | T | ALT | REF | REF | REF | REF | REF |
| 4021177 | C | T | REF | REF | REF | REF | REF | ALT |
| 4028111 | T | C | REF | REF | ALT | REF | REF | REF |
| 4040114 | G | A | REF | REF | REF | REF | ALT | REF |
| 4042189 | T | C | REF | REF | ALT | REF | REF | REF |
| 4045487 | A | G | ALT | ALT | ALT | ALT | ALT | ALT |
| 4096077 | C | T | ALT | ALT | ALT | ALT | ALT | ALT |
| 4114396 | T | C | REF | REF | ALT | REF | REF | REF |
| 4115033 | T | C | REF | ALT | REF | ALT | REF | ALT |
| 4141179 | C | T | REF | REF | REF | REF | ALT | REF |
| 4175718 | G | A | ALT | REF | ALT | REF | ALT | REF |
| 4184624 | G | A | ALT | REF | REF | REF | REF | REF |
| 4206587 | C | T | ALT | ALT | ALT | ALT | ALT | ALT |
| 4224084 | T | C | ALT | REF | REF | REF | REF | REF |
| 4283145 | C | A | ALT | REF | REF | REF | REF | REF |
| 4296994 | A | G | ALT | ALT | ALT | ALT | ALT | ALT |
| 4305456 | C | G | ALT | REF | REF | REF | REF | REF |
| 4310338 | A | G | ALT | REF | REF | REF | REF | REF |
| 4349421 | C | G | ALT | REF | REF | REF | REF | REF |
| 4371206 | A | G | ALT | REF | REF | REF | REF | REF |
| 4394282 | A | G | ALT | ALT | ALT | ALT | ALT | ALT |
| 4434621 | C | T | ALT | REF | REF | REF | REF | REF |
| 4456097 | T | C | ALT | REF | REF | REF | REF | REF |
| 4471234 | A | G | REF | REF | ALT | REF | REF | REF |
| 4523508 | T | C | ALT | REF | REF | REF | REF | REF |
| 4560344 | T | C | ALT | ALT | ALT | ALT | ALT | ALT |
| 4568049 | G | A | REF | REF | ALT | REF | REF | REF |
| 4588924 | C | G | ALT | REF | REF | REF | ALT | REF |
| 4610094 | G | A | REF | ALT | REF | ALT | REF | ALT |
| 4610738 | A | G | ALT | REF | REF | REF | REF | REF |
| 4623641 | A | C | REF | REF | REF | REF | ALT | REF |
| 4636226 | C | T | ALT | REF | REF | REF | REF | REF |
| 4652218 | G | A | REF | REF | ALT | REF | REF | REF |
| 4661771 | C | T | REF | ALT | REF | ALT | REF | ALT |
| 4664194 | C | T | REF | REF | REF | REF | ALT | REF |
| 4668391 | C | T | ALT | REF | REF | REF | REF | REF |
| 4678708 | C | G | REF | REF | REF | REF | ALT | REF |
| 4704217 | G | A | REF | REF | REF | REF | ALT | REF |
| 4731216 | T | C | ALT | REF | REF | REF | REF | REF |
| 4736169 | A | G | ALT | REF | REF | REF | REF | REF |
| 4775418 | G | A | ALT | REF | REF | REF | REF | REF |
| 4776431 | G | C | REF | REF | ALT | REF | REF | REF |
| 4782828 | T | C | ALT | REF | REF | REF | REF | REF |
| 4804579 | G | C | REF | ALT | REF | ALT | REF | ALT |
| 4830428 | A | G | ALT | REF | REF | REF | REF | REF |

**^a^** The numerical position of the respective nucleotide corresponds to that present in the revised genome sequence of the K-10 strain in the public database (Wynne *et al*., 2010).

**^b^** The identity of the nucleotide at that specific locus was the same as the reference K-10 strain.

**^c^** The identity of the nucleotide at that specific locus was different from the reference K-10.

**^b,c^** The identities of the specific nucleotides at the corresponding positions in each isolate are also indicated.
