# Supplementary material for: Examination of Mycobacterium avium subspecies paratuberculosis mixed genotype infections in dairy animals using a whole genome sequencing approach
Source: PeerJ. 2016 Dec 14;4:e2793. doi: 10.7717/peerj.2793 (PMC5160890; doi:10.7717/peerj.2793)
Supplement: Table S2 — Details regarding SNP identification and analysis are described in the text of the manuscript. [file peerj-04-2793-s003.docx]

**Supplementary Table 2.** Distance matrix based on pairwise SNP differences between NL *Map* isolates, representative Canadian isolates and the reference K-10 strain.

| **Strain/**  **Isolate** |  | **1** | **2** | **3** | **4** | **5** | **6** | **7** | **8** | **9** | **10** | **11** | **12** | **13** |
| --- | --- | --- | --- | --- | --- | --- | --- | --- | --- | --- | --- | --- | --- | --- |
| **89C** | **1** | — | 226 | 233 | 226 | 207 | 224 | 216 | 643 | 11 | 221 | 207 | 246 | 198 |
| **93B** | **2** | 226 | — | 125 | 8 | 115 | 6 | 65 | 642 | 225 | 224 | 208 | 244 | 74 |
| **95A** | **3** | 233 | 125 | — | 125 | 120 | 123 | 115 | 646 | 232 | 229 | 213 | 248 | 95 |
| **95B** | **4** | 226 | 8 | 125 | — | 115 | 4 | 65 | 642 | 225 | 224 | 208 | 244 | 74 |
| **95E** | **5** | 207 | 115 | 120 | 115 | — | 113 | 105 | 625 | 206 | 203 | 187 | 225 | 85 |
| **96E** | **6** | 224 | 6 | 123 | 4 | 113 | — | 63 | 640 | 223 | 222 | 206 | 242 | 72 |
| **A1_067 ^a^** | **7** | 216 | 65 | 115 | 65 | 105 | 63 | — | 630 | 215 | 213 | 198 | 234 | 64 |
| **A1_075  ^a^** | **8** | 643 | 642 | 646 | 642 | 625 | 640 | 630 | — | 642 | 632 | 618 | 659 | 612 |
| **A1_092  ^a^** | **9** | 11 | 225 | 232 | 225 | 206 | 223 | 215 | 642 | — | 220 | 206 | 245 | 197 |
| **A1_139  ^a^** | **10** | 221 | 224 | 229 | 224 | 203 | 222 | 213 | 632 | 220 | — | 201 | 241 | 194 |
| **A1_194 ^a^** | **11** | 207 | 208 | 213 | 208 | 187 | 206 | 198 | 618 | 206 | 201 | — | 224 | 178 |
| **A1_377  ^a^** | **12** | 246 | 244 | 248 | 244 | 225 | 242 | 234 | 659 | 245 | 241 | 224 | — | 214 |
| **Ref_K10 ^b^** | **13** | 198 | 74 | 95 | 74 | 85 | 72 | 64 | 612 | 197 | 194 | 178 | 214 | — |

**^a^** Isolates previously sequenced that are representative of different *Map* clades from Canada (Ahlstrom *et al*., 2016).

^b^ The revised (Wynne *et al*., 2010) genome sequence of the K10 strain in the public database was used for comparison.
